# Prospective Study on Sonographic Measurement of Umbilical Cord Thickness, Fetal Fat Layer, and Interventricular Septal Thickness as Predictors of Macrosomia in Fetuses of Women With Gestational Diabetes Mellitus

**DOI:** 10.7759/cureus.84198

**Published:** 2025-05-15

**Authors:** Dirisala Anudeep, Shivanand Pati, Siddaroodha Sajjan, Divyashree Koppal, Pavan Kolekar

**Affiliations:** 1 Radiodiagnosis, Shri B. M. Patil Medical College, Hospital and Research Centre, Bijapur Liberal District Educational (BLDE) Association (Deemed to Be University), Vijayapura, IND

**Keywords:** fetal fat layer, fetal macrosomia, gestational diabetes mellitus, interventricular septal thickness, pregnancy complications, sonography, umbilical cord thickness

## Abstract

Introduction: Fetal macrosomia is a common and concerning complication of gestational diabetes mellitus (GDM), associated with increased risks for both maternal and neonatal morbidity. Traditional methods of predicting macrosomia often lack precision, particularly in diabetic pregnancies. This study aimed to evaluate the efficacy of three sonographic parameters, umbilical cord thickness (UCT), fetal fat layer (FFL), and interventricular septal thickness (IVS), as predictors of fetal macrosomia in women with GDM.

Materials and methods: This prospective study included 123 pregnant women with GDM between 34 and 40 weeks of gestation. Comprehensive maternal data, including body mass index (BMI) and glycemic parameters (fasting blood sugar (FBS), postprandial blood sugar (PPBS), and glycated hemoglobin (HbA1c)), were recorded. Sonographic measurements of UCT, FFL, and IVS were performed and analyzed for their association with birth outcomes. Macrosomia was defined as a birth weight greater than 4000 g.

Results: Macrosomia occurred in 62.6% of pregnancies, with strong associations with maternal BMI (p<0.001) and HbA1c levels (p<0.001). Sonographic parameters showed significant correlations with birth weight: UCT (r=0.792, p<0.001), FFL (r=0.34, p<0.001), and IVS (r=0.295, p=0.001). A UCT ≥25 mm demonstrated excellent sensitivity (93.3%) and specificity (85.4%) for predicting macrosomia. FFL ≥4.5 mm showed high specificity (93.3%) and positive predictive value (PPV) (97.3%), while IVS ≥3.9 mm exhibited good specificity (85%) but lower sensitivity (71.8%). Despite the high prevalence of macrosomia, 88.6% of deliveries were uncomplicated, though the cesarean section rate was high (64.2%).

Conclusion: Sonographic measurements of UCT, FFL, and IVS are valuable predictors of fetal macrosomia in GDM pregnancies. UCT, in particular, demonstrated the strongest correlation with birth weight and superior diagnostic accuracy. The integration of these sonographic parameters with maternal factors can enhance the accuracy of macrosomia prediction, potentially improving clinical decision-making and optimizing maternal and neonatal outcomes.

## Introduction

Gestational diabetes mellitus (GDM) affects approximately 7-25% of pregnancies worldwide, with prevalence varying widely depending on the population studied and the diagnostic criteria employed [1]. It is a carbohydrate intolerance disorder that is first recognized during pregnancy and is associated with a range of adverse maternal and fetal outcomes. Among these, fetal macrosomia is one of the most significant and common complications. Defined as a birth weight exceeding 4000 g or above the 90th percentile for gestational age, macrosomia occurs in 15-45% of diabetic pregnancies, in stark contrast to approximately 10% in the general population [2,3].

Accurate prenatal prediction of macrosomia remains a critical yet unresolved challenge in obstetric practice, especially in pregnancies complicated by GDM. Traditional approaches such as clinical estimation of fetal weight and standard ultrasonographic biometry (including biparietal diameter, abdominal circumference, and femur length) are commonly used. However, these methods demonstrate only moderate predictive capability, with reported sensitivities ranging between 50% and 75%, and are often less reliable in GDM pregnancies due to the unique fetal growth patterns observed [4,5].

In response to these limitations, attention has shifted toward alternative sonographic markers that might more accurately reflect the metabolic effects of maternal hyperglycemia on the fetus. Recent studies have highlighted several novel parameters, including umbilical cord thickness (UCT), which may correlate with increased Wharton's jelly deposition seen in diabetic pregnancies; fetal fat layer (FFL) thickness, a direct measure of excess subcutaneous adiposity in infants of diabetic mothers; and interventricular septal thickness (IVS), which may reflect fetal myocardial hypertrophy secondary to intrauterine hyperinsulinemia [3,4,6-8].

These markers offer potential improvements in both sensitivity and specificity for predicting macrosomia in GDM pregnancies. However, existing literature remains limited and lacks consensus on their combined diagnostic value. Therefore, the present prospective study aims to evaluate the predictive accuracy of UCT, FFL, and IVS, individually and in combination, for identifying macrosomia in fetuses of women diagnosed with GDM.

## Materials and methods

This prospective observational study was conducted over a period of two years from April 2023 to April 2025 at Shri B.M. Patil Medical College and Hospital, Vijayapura, following approval from the Institutional Ethics Committee (BLDE/IEC/941/2023-24). Informed written consent was obtained from all participants prior to enrolment. The study included 123 pregnant women diagnosed with GDM, with singleton pregnancies beyond 27 weeks and six days of gestation, and confirmed the presence of a three-vessel umbilical cord on ultrasonography. Women were excluded if they had a gestational age of less than 27 weeks and six days, if they had multiple gestations, or if they were either overtly diabetic (pre-existing diabetes) or did not have GDM. The estimated sample size for this study was calculated to be 123, using the formula n=(Z²×p×(1-p))/d² based on an assumed macrosomia prevalence of 8.75% among pregnant women with GDM, with a 95% confidence level and 5% margin of error.

Baseline demographic and clinical data including maternal age, body mass index (BMI), parity, and glycemic parameters, namely fasting blood sugar (FBS), postprandial blood sugar (PPBS), and glycated hemoglobin (HbA1c), were recorded at the time of enrolment. Sonographic examinations were performed using GE Voluson S8 BT18 and GE Versana Premier ultrasound machines equipped with 4-8 MHz transabdominal probes. All scans were conducted by certified sonographers, including radiologists and obstetricians with formal ultrasound training, who were blinded to the participants’ glycemic profiles to eliminate potential bias.

The study focused on three key sonographic parameters: UCT, FFL, and IVS. UCT was measured at the mid-portion of the cord in a cross-sectional view, perpendicular to its long axis. Fetal fat thickness was assessed at three standardized anatomical locations: the anterior abdominal wall, mid-thigh, and mid-arm. IVS was measured in the four-chamber cardiac view during diastole. Each of these parameters was measured three times per examination, and the average of the readings was used for analysis to improve reliability. Serial sonographic assessments were performed at four-week intervals until delivery, during which participants continued routine antenatal follow-up every two weeks.

**Figure 1:**
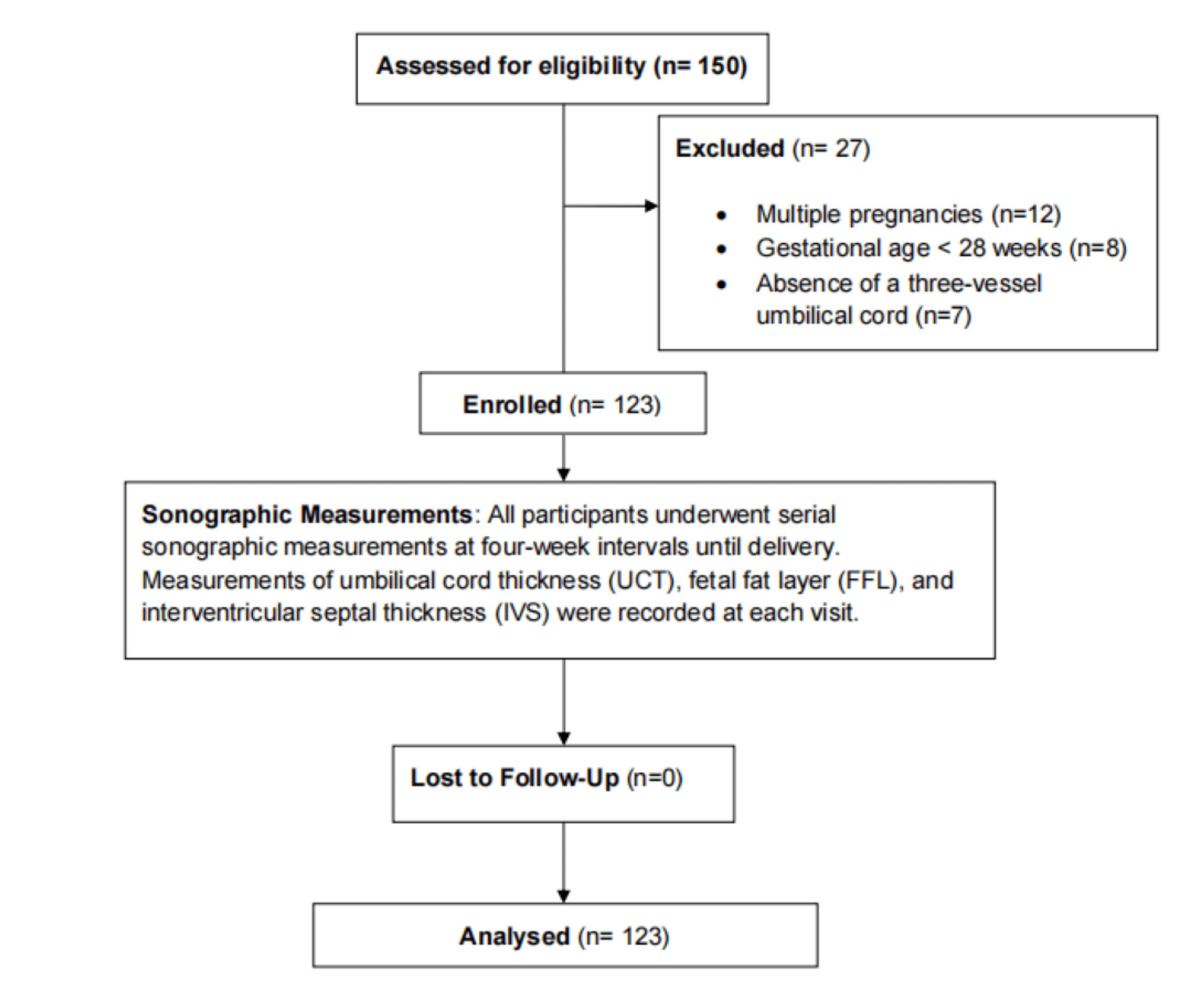
Flow chart

Following delivery, neonatal birth weight was recorded using calibrated electronic scales. Macrosomia was defined as a birth weight exceeding 4000 g. Data were entered and analyzed using SPSS version 21 (IBM Corp., Armonk, New York, USA). Categorical variables were expressed as frequencies and percentages, while continuous variables were summarized using mean and standard deviation. Student’s t-test and chi-square test were employed to assess statistical differences between macrosomic and non-macrosomic groups, with a p-value of less than 0.05 considered statistically significant (Figure 1).

## Results

A total of 123 pregnant women with GDM were included in the study. The majority of participants were aged 20-30 years (69 (56.1%)), followed by those aged 31-40 years (54 (43.9%)). Most participants were at a gestational age of 37-40 weeks (68 (55.3%)), while 55 (44.7%) were between 34 and 36 weeks. Based on BMI, 59 (48%) were overweight (25-29.9 kg/m²), 36 (29.3%) were obese (>30 kg/m²), and 28 (22.8%) had a normal BMI (18.5-24.9 kg/m²). Mean fasting blood sugar was 113.3±18.1 mg/dL, postprandial sugar was 190.1±35.8 mg/dL, and HbA1c was 6.33±0.72% (Table 1).

**Table 1:** Demographic and clinical characteristics Categorical variables were expressed as frequencies and percentages, while continuous variables were summarized using mean and standard deviation. FBS, fasting blood sugar; PPBS, postprandial blood sugar; HbA1c, glycated hemoglobin; BMI, body mass index

Parameter	Category/Measurement	Frequency/Value
Age (years)	20-30	69 (56.1%)
	31-40	54 (43.9%)
Gestational age (weeks)	34-36	55 (44.7%)
	37-40	68 (55.3%)
BMI (kg/m^2^)	18.5-24.9	28 (22.8%)
	25-29.9	59 (48%)
	>30	36 (29.3%)
Blood sugar levels	FBS (mean±SD)	113.3±18.1 mg/dL
	PPBS (mean±SD)	190.1±35.8 mg/dL
	HbA1c (mean±SD)	6.33±0.72%

**Figure 2:**
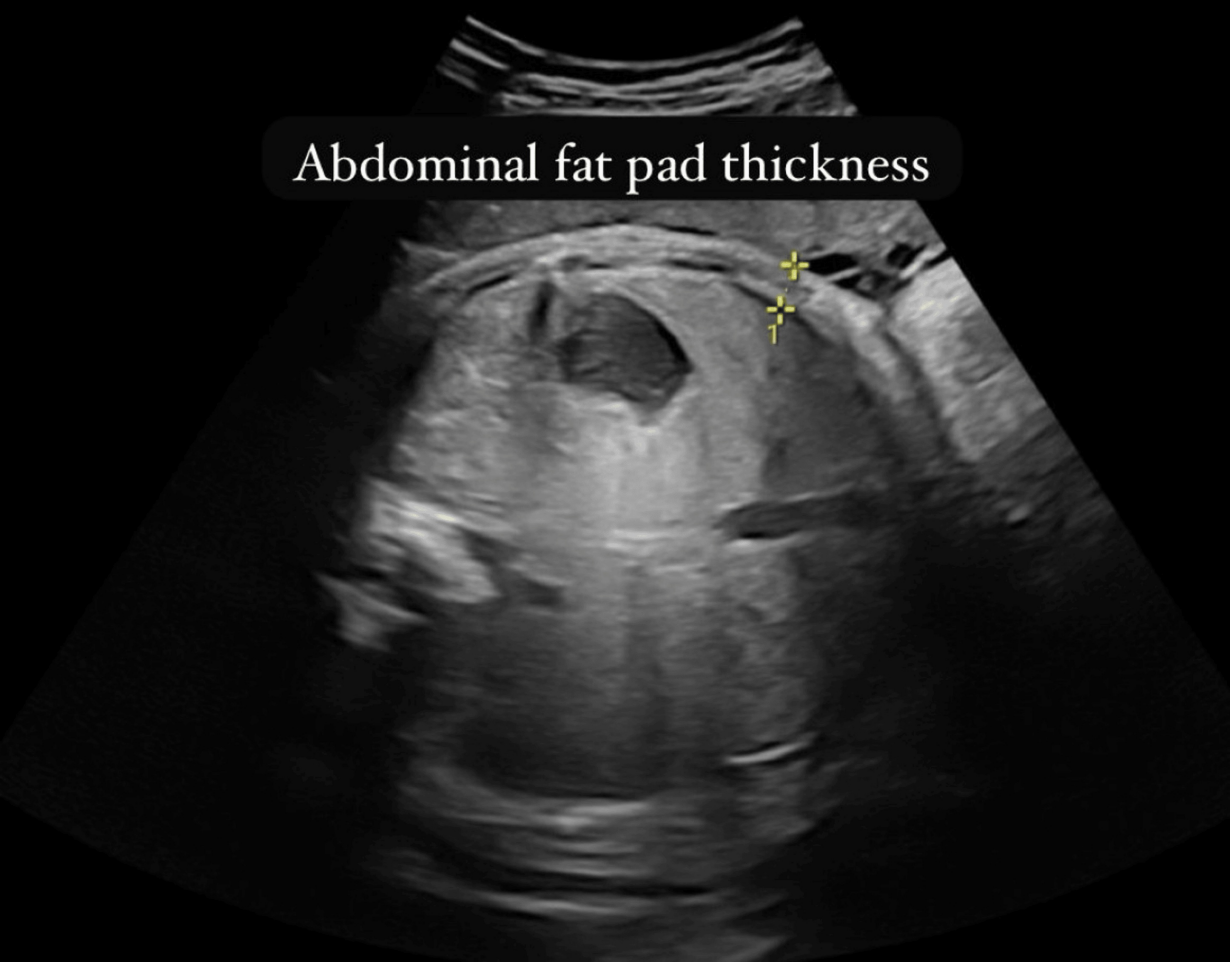
Abdominal fat pad thickness

**Figure 3:**
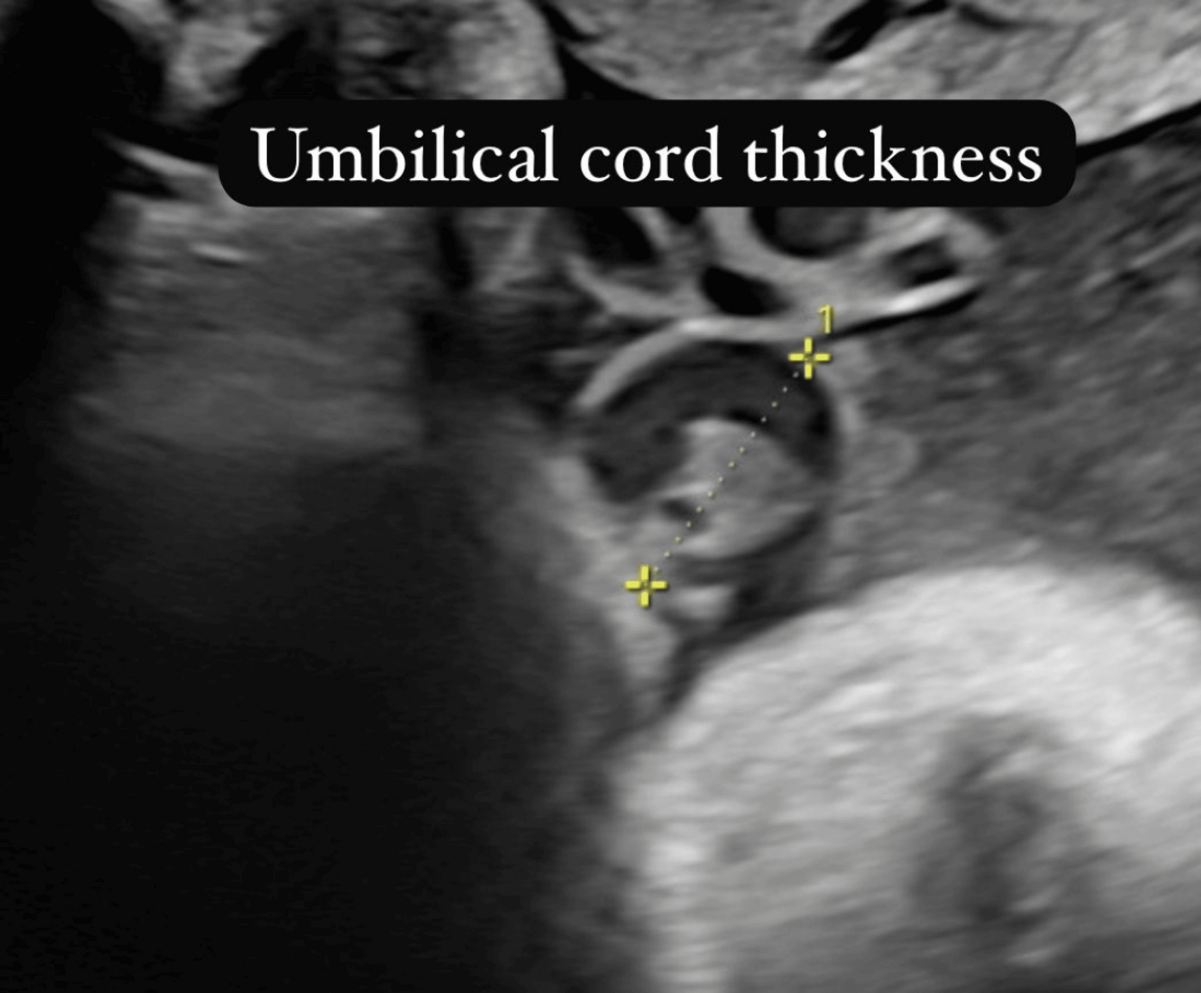
Umbilical cord thickness

**Figure 4:**
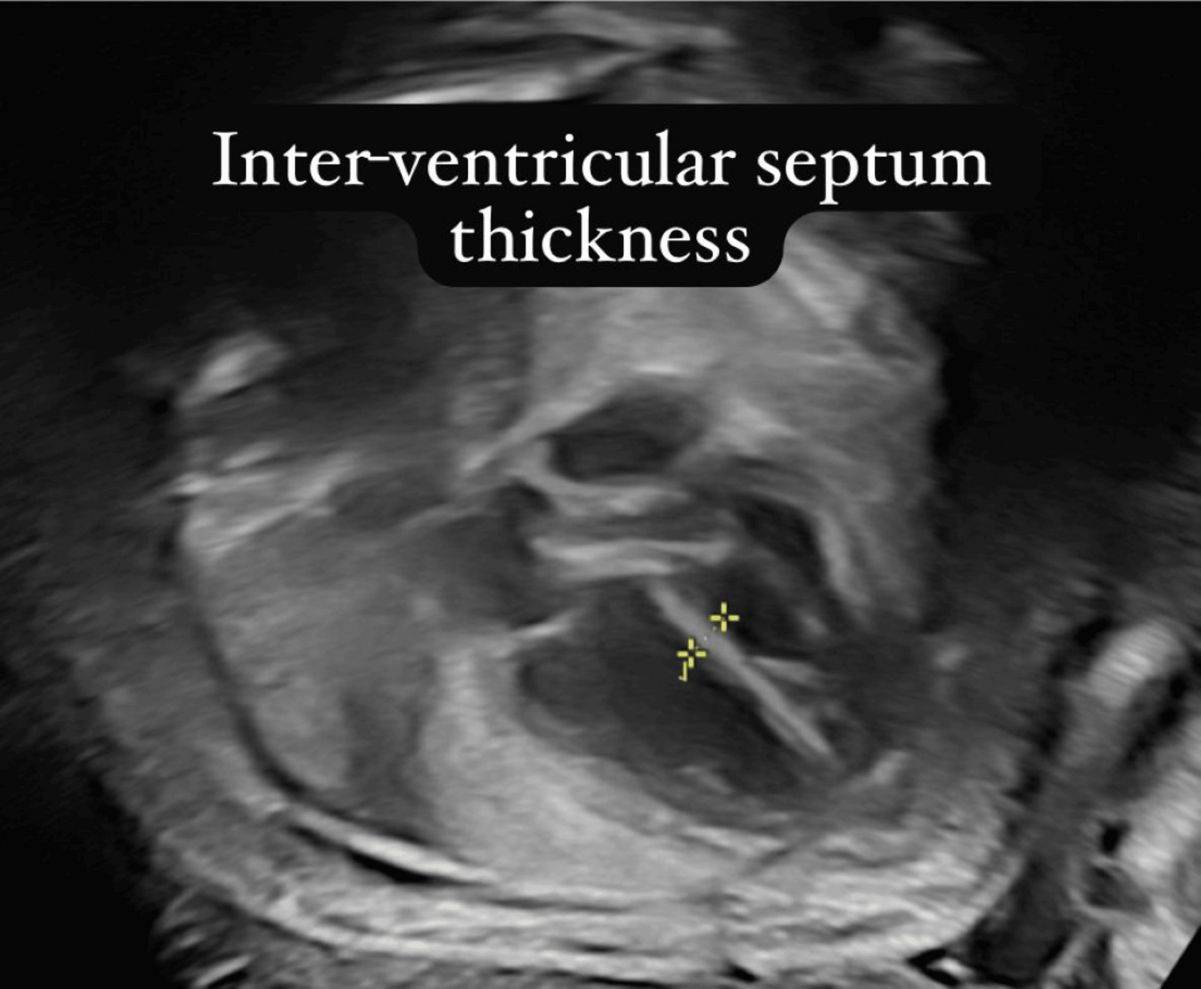
Interventricular septum thickness

The sonographic evaluation revealed a mean UCT of 23.2±6.9 mm, FFL of 5.21±1.2 mm, and IVS of 5.1±1.33 mm. Regarding birth outcomes, 47 (38.2%) neonates were macrosomic (>4000 g), 75 (61%) had normal birth weight (2500-4000 g), and one (0.8%) had low birth weight (<2500 g). Vaginal delivery was performed in 44 (35.8%) cases, whereas 79 (64.2%) underwent lower segment cesarean section (LSCS). Complications included birth injuries in seven (5.7%), shoulder dystocia in six (4.9%), and meconium aspiration in one (0.8%), while 109 (88.6%) had no complications (Table 2; Figure 2, Figure 3, and Figure 4).

**Table 2:** Sonographic measurements and birth outcomes Categorical variables were expressed as frequencies and percentages, while continuous variables were summarized using mean and standard deviation. LSCS, lower segment cesarean section

Parameter	Category/Measurement	Frequency/Value
Sonographic measurements	Umbilical cord thickness (mean±SD)	23.2±6.9 mm
	Fetal fat layer (mean±SD)	5.21±1.2 mm
	Interventricular septal thickness (mean±SD)	5.1±1.33 mm
Birth weight (grams)	<2500	1 (0.8%)
	2500-4000	75 (61%)
	>4000	47 (38.2%)
Macrosomia	Present	47 (38.2%)
	Absent	76 (61.8%)
Mode of delivery	Vaginal	44 (35.8%)
	LSCS	79 (64.2%)
Complications	Birth injury	7 (5.7%)
	Meconium aspiration	1 (0.8%)
	Shoulder dystocia	6 (4.9%)
	None	109 (88.6%)

Significant associations were observed between macrosomia and various maternal and fetal parameters. Among women with macrosomic neonates, 30 (39%) had a BMI >30, 40 (51.9%) had a BMI of 25-29.9, and seven (9.1%) had a BMI of 18.5-24.9, compared to six (13%), 19 (41.3%), and 21 (45.7%), respectively, in the non-macrosomic group (χ²=24.1986, p<0.001). The mean HbA1c in the macrosomic group was 6.72±0.44% compared to 5.68±0.63% in the non-macrosomic group (t=9.86, p<0.001). UCT ≥25 mm was found in 67 (87%) of macrosomic fetuses, compared to four (8.7%) in the non-macrosomic group (χ²=72.3781, p<0.001). An FFL ≥4.5 mm was observed in 73 (94.8%) macrosomic fetuses, compared to 17 (37%) in the non-macrosomic group (χ²=49.0889, p<0.001). Similarly, an IVS ≥3.9 mm was seen in 74 (96.1%) macrosomic cases, versus 29 (63%) in the non-macrosomic group (χ²=23.1154, p<0.001) (Table 3).

**Table 3:** Association of macrosomia with maternal factors and sonographic parameters Categorical variables were expressed as frequencies and percentages, while continuous variables were summarized using mean and standard deviation. Student’s t-test and chi-square test were employed to assess statistical differences. A p-value of less than 0.05 was considered statistically significant. HbA1c, glycated hemoglobin; BMI, body mass index

Parameter		Macrosomia		Statistical Value	p-value
		Absent	Present		
BMI (kg/m^2^)	18.5-24.9	21 (45.7%)	7 (9.1%)	24.1986 (Chi-square value)	<0.001
	25-29.9	19 (41.3%)	40 (51.9%)		
	>30	6 (13%)	30 (39%)		
HbA1c	(mean±SD)	5.68±0.63	6.72±0.44	9.86 (t-value)	<0.001
Umbilical cord thickness	<25 mm	42 (91.3%)	10 (13%)	72.3781 (Chi-square value)	<0.001
	≥25 mm	4 (8.7%)	67 (87%)		
Fetal fat layer	<4.5 mm	29 (63%)	4 (5.2%)	49.0889 (Chi-square value)	<0.001
	≥4.5 mm	17 (37%)	73 (94.8%)		
Interventricular septal thickness	<3.9 mm	17 (37%)	3 (3.9%)	23.1154 (Chi-square value)	<0.001
	≥3.9 mm	29 (63%)	74 (96.1%)		

Diagnostic accuracy analysis showed that UCT >25 mm had the highest sensitivity (93.3%) and high specificity (85.4%), with a positive predictive value (PPV) of 90% and a negative predictive value (NPV) of 89%. FFL >4.5 mm demonstrated a sensitivity of 80.2%, specificity of 93.3%, PPV of 97.3%, and NPV of 60.8%. IVS >3.9 mm yielded a sensitivity of 71.8%, specificity of 85%, PPV of 96.1%, and NPV of 36.9% (Table 4).

**Table 4:** Diagnostic accuracy of sonographic parameters for predicting macrosomia PPV, positive predictive value; NPV, negative predictive value

Parameter	Sensitivity	Specificity	PPV	NPV
Umbilical cord thickness (>25 mm)	93.3%	85.4%	90%	89%
Fetal fat layer (>4.5 mm)	80.2%	93.3%	97.3%	60.8%
Interventricular septal thickness (>3.9 mm)	71.8%	85%	96.1%	36.9%

## Discussion

Our study demonstrates significant correlations between sonographic parameters and macrosomia in pregnancies complicated by GDM. Notably, UCT showed the strongest correlation with birth weight (r=0.792, p<0.001) and exhibited excellent sensitivity (93.3%) and specificity (85.4%) at a cut-off of ≥25 mm. These findings are consistent with those of Cromi et al., who reported that a larger umbilical cord cross-sectional area was significantly associated with macrosomia, with sensitivity and specificity values of 90.9% and 83.6%, respectively [9]. Likewise, Raio et al. demonstrated that UCT is increased in diabetic pregnancies, correlating with maternal glycemic control and fetal hyperinsulinemia [10]. The underlying physiological mechanism may involve the increased deposition of Wharton's jelly in the umbilical cord, which serves to protect the umbilical vessels from mechanical compression while also reflecting the altered metabolic environment in diabetic pregnancies.

FFL thickness ≥4.5 mm showed high specificity (93.3%) and PPV (97.3%) for macrosomia, with 94.8% of macrosomic fetuses exhibiting fat layer measurements above this threshold. These results are in line with those of Seth et al., who observed that fetal abdominal subcutaneous tissue thickness >5 mm at 28-32 weeks was associated with a 3.2-fold increased risk of macrosomia [11]. Additionally, Larciprete et al. demonstrated that fetal fat measurements in the abdomen, arm, and thigh were significantly greater in diabetic pregnancies, with abdominal fat showing the strongest correlation with birth weight (r=0.61) [12]. The physiological basis for these findings aligns with the Pedersen hypothesis, which postulates that maternal hyperglycemia induces fetal hyperinsulinemia, leading to an increase in fetal fat deposition, particularly in the abdominal region [13].

IVS ≥3.9 mm, while exhibiting a lower sensitivity (71.8%), demonstrated good specificity (85%) for predicting macrosomia. Similar findings were reported by Garcia-Flores et al., who found that IVS ≥3.9 mm was a predictor of macrosomia, with a sensitivity of 84.2% and specificity of 64.2% [14]. Lisowski et al. provided insight into the physiological mechanisms, showing that fetal hyperinsulinemia directly stimulates cardiac myocyte growth through insulin-like growth factor pathways [15].

Incorporating these sonographic parameters, along with maternal factors such as BMI and glycemic control, may offer a more comprehensive and reliable approach to predicting macrosomia compared to relying on any single measurement alone. This approach is supported by Maruotti et al., who integrated multiple predictors, resulting in an improved area under the receiver operating characteristic (ROC) curve of 0.92, compared to 0.74 for standard biometry alone [16]. Thus, the combination of sonographic markers and maternal characteristics may enhance the accuracy of macrosomia prediction and guide clinical decision-making in the management of pregnancies complicated by GDM.

The strengths of this study include the use of multiple sonographic parameters and a specific focus on pregnancies complicated by GDM, a high-risk population in which accurate prediction of macrosomia is particularly valuable. The evaluation of actual birth outcomes and complications further provided important clinical context for the sonographic findings.

However, several limitations must be acknowledged. First, the sample size of 123 pregnancies, while adequate for exploratory analysis, may limit the statistical power for detecting less frequent outcomes and restrict the generalizability of findings to broader populations. Second, the study did not directly compare the diagnostic performance of the three sonographic parameters (UCT, FFL, and IVS) with standard fetal biometric indices such as abdominal circumference or estimated fetal weight. Such a comparison could have provided valuable insight into the incremental utility of these novel parameters. Third, the study did not evaluate how incorporating these sonographic markers into routine clinical decision-making might influence maternal or neonatal outcomes, an important area that warrants prospective investigation.

## Conclusions

This prospective study confirms that sonographic measurements of UCT, FFL, and IVS are effective predictors of fetal macrosomia in pregnancies complicated by GDM. Among these, UCT demonstrated the strongest correlation with birth weight and provided superior diagnostic accuracy, with excellent sensitivity (93.3%) and specificity (85.4%) at a cut-off value of ≥25 mm. The study also emphasizes the significant role of maternal factors, such as BMI and glycemic control, in determining the risk of macrosomia. Despite the high prevalence of macrosomia (62.6%) in our study cohort, the majority of deliveries (88.6%) were uncomplicated, indicating that with proper antenatal risk assessment and management, complications associated with fetal macrosomia can be effectively mitigated. The incorporation of these sonographic parameters into routine clinical practice can enhance risk stratification, improve obstetric decision-making, and potentially reduce perinatal morbidity, thereby optimizing maternal and neonatal outcomes.

## References

[1] Plows JF, Stanley JL, Baker PN, Reynolds CM, Vickers MH (2018). The pathophysiology of gestational diabetes mellitus. Int J Mol Sci.

[2] Metzger BE, Lowe LP, Dyer AR (2008). Hyperglycemia and adverse pregnancy outcomes. N Engl J Med.

[3] Matsumoto M, Yanagihara T, Hata T (2000). Three-dimensional qualitative sonographic evaluation of fetal soft tissue. Hum Reprod.

[4] Bethune M, Bell R (2003). Evaluation of the measurement of the fetal fat layer, interventricular septum and abdominal circumference percentile in the prediction of macrosomia in pregnancies affected by gestational diabetes. Ultrasound Obstet Gynecol.

[5] Melamed N, Yogev Y, Meizner I, Mashiach R, Ben-Haroush A (2010). Sonographic prediction of fetal macrosomia: the consequences of false diagnosis. J Ultrasound Med.

[6] Elgendy H, Tarek A, Shahat M (2025). A prospective cross-sectional study for prediction of fetal macrosomia using sonographic measurement of placental thickness and umbilical cord cross-section area. Hellenic J Obstet Gynecol.

[7] Figueroa H, Silva MC, Kottmann C (2012). Fetal evaluation of the modified-myocardial performance index in pregnancies complicated by diabetes. Prenat Diagn.

[8] Huang P, Deng Y, Feng L, Gao Y, Cheng X, Liu H (2023). Evaluation of fetal cardiac function in maternal gestational diabetes mellitus by speckle-tracking echocardiography. J Ultrasound Med.

[9] Cromi A, Ghezzi F, Di Naro E, Siesto G, Bergamini V, Raio L (2007). Large cross-sectional area of the umbilical cord as a predictor of fetal macrosomia. Ultrasound Obstet Gynecol.

[10] Raio L, Ghezzi F, Di Naro E, Duwe DG, Cromi A, Schneider H (2003). Umbilical cord morphologic characteristics and umbilical artery Doppler parameters in intrauterine growth-restricted fetuses. J Ultrasound Med.

[11] Seth I, Aiyappan RK, Singh S (2023). Mid-trimester fetal anterior abdominal wall subcutaneous tissue thickness: an early ultrasonographic predictor of gestational diabetes mellitus. Cureus.

[12] Larciprete G, Valensise H, Vasapollo B (2003). Fetal subcutaneous tissue thickness (SCTT) in healthy and gestational diabetic pregnancies. Ultrasound Obstet Gynecol.

[13] Catalano PM, McIntyre HD, Cruickshank JK (2012). The hyperglycemia and adverse pregnancy outcome study: associations of GDM and obesity with pregnancy outcomes. Diabetes Care.

[14] Garcia-Flores J, Jañez M, Gonzalez MC, Martinez N, Espada M, Gonzalez A (2011). Fetal myocardial morphological and functional changes associated with well-controlled gestational diabetes. Eur J Obstet Gynecol Reprod Biol.

[15] Lisowski LA, Verheijen PM, Copel JA, Kleinman CS, Wassink S, Visser GH, Meijboom EJ (2010). Congenital heart disease in pregnancies complicated by maternal diabetes mellitus. An international clinical collaboration, literature review, and meta-analysis. Herz.

[16] Maruotti GM, Saccone G, Martinelli P (2017). Third trimester ultrasound soft-tissue measurements accurately predicts macrosomia. J Matern Fetal Neonatal Med.

